# A Comparison Between Cefepime and Piperacillin-Tazobactam in the Management of Septic Shock

**DOI:** 10.7759/cureus.18742

**Published:** 2021-10-13

**Authors:** Robert C Ross, Abbie N Rosen, Kenneth K Tran, Katharyn L Smith, Andrew J Franck

**Affiliations:** 1 Pharmacy, North Florida/South Georgia Veterans Health System, Gainesville, USA; 2 Pharmacy, Dana-Farber Cancer Institute, Boston, USA; 3 Pharmacy, University of Iowa Hospitals & Clinics, Iowa City, USA

**Keywords:** acute kidney injury, sepsis, infectious diseases, anti-infectives, critical care

## Abstract

Introduction

Septic shock is defined as a dysregulated host response to infection characterized by hemodynamic instability. Concern for the increased risk of acute kidney injury (AKI) with piperacillin-tazobactam in combination with vancomycin may prompt more use of alternative broad-spectrum antipseudomonal beta-lactam antibiotics, such as cefepime. This study assessed whether cefepime was associated with improved outcomes compared to piperacillin-tazobactam in patients with septic shock.

Methods

This retrospective cohort study included veterans treated for septic shock between September 1, 2008, and August 31, 2018. This study compared cefepime and piperacillin-tazobactam as initial antibiotic management for septic shock. Outcomes included AKI, *Clostridioides difficile* infection (CDI), hospital length of stay, intensive care unit mortality, and mortality within 30 days of hospitalization.

Results

In total, 240 patients were included in this study (120 in each cohort). The proportion of AKI was 60.0% in the piperacillin-tazobactam cohort compared to 58.3% in the cefepime cohort (p = 0.90). Mortality was significantly higher in the cefepime cohort. There were no significant differences in CDI or hospital length of stay.

Conclusion

The results of this study do not suggest that the use of the antipseudomonal beta-lactam antibiotic used in the initial management of septic shock is associated with differences in the AKI or CDI. The higher mortality observed with cefepime may warrant further investigation.

## Introduction

Sepsis, which affects 1.7 million Americans yearly with approximately 270,000 cases resulting in death, is defined as a life-threatening organ dysfunction caused by an imbalanced response to infection [1-3]. Septic shock is a subset of sepsis with a greater risk of mortality, identified by refractory hypotension. Specifically, septic shock is defined by a vasopressor requirement to maintain a mean arterial pressure (MAP) of 65 mmHg or greater and a serum lactate level greater than 2 mmol/L in the absence of hypovolemia [1,3]. Although the management of septic shock has evolved and is periodically updated, antimicrobials remain a mainstay of appropriate sepsis management. Current guidelines recommend rapid initiation of empiric broad-spectrum antimicrobial therapy to target common gram-negative and gram-positive bacteria. Thus, initial antimicrobial regimens frequently include the antipseudomonal beta-lactam antibiotics cefepime or piperacillin-tazobactam with further selection requiring consideration of the source of infection and several other patient-specific factors [1,3]. One important consideration in antibiotic selection is the risk of adverse events such as nephrotoxicity.

In recent years, several studies have examined the potential nephrotoxicity of piperacillin-tazobactam when used in combination with vancomycin and have shown benefits of using cefepime in place of piperacillin-tazobactam to reduce the incidence of acute kidney injury (AKI); however, most of these studies in the critical care setting have failed to observe a difference in the proportion of AKI between piperacillin-tazobactam and cefepime [4-8]. Factors identified to increase the risk of AKI and nephrotoxicity in the critically ill are a vancomycin daily dose greater than 4 g, serum trough levels greater than 15 mg/mL, and a duration of therapy of greater than seven days [4,5]. The addition of vancomycin to piperacillin-tazobactam therapy can increase, or even double as shown in some studies, the incidence of AKI compared to cefepime [4,5,9,10]. Overall, these studies show a trend towards an increased risk of AKI when using piperacillin-tazobactam; however, the margin of difference appears significantly smaller in the critical care setting. Furthermore, no studies have evaluated the risk of piperacillin-tazobactam nephrotoxicity for patients specifically with septic shock.

Cefepime has been associated with adverse neurologic effects and an increased incidence of hospital-acquired *Clostridioides difficile* infection (CDI) [11,12]. Susceptibility to infection by *C. difficile* is induced when certain factors, most often antibiotics, disrupt the gut microflora [11]. Infection prevention measures and antimicrobial stewardship programs have been implemented to choose the most appropriate antibiotic with the narrowest spectrum to help decrease CDI [12]. A meta-analysis comparing antibiotic classes and their association with hospital-acquired CDI found third-generation cephalosporins to have the highest odds, followed by clindamycin, second-generation cephalosporins, and fourth-generation cephalosporins [11]. One study found a significantly increased rate of CDI when changing empiric therapy from meropenem to cefepime; however, the patients were immunocompromised at baseline, so further studies would be needed to determine this association [12]. Interestingly, piperacillin-tazobactam may have some protection against CDI during the course of antibiotic therapy [13].

While there is consensus on the benefits of early, broad antimicrobial coverage, it remains unclear if one antimicrobial agent should be preferred during the initial management of septic shock. To determine whether the choice of antipseudomonal beta-lactam antibiotic in the initial management of septic shock was associated with any benefit, this study was conducted to compare the impact of the use of intravenous (IV) cefepime with piperacillin-tazobactam on observed patient outcomes over a 10-year period, including the incidence of AKI, CDI, hospital length of stay and mortality. Portions of this research were previously presented as a meeting abstract at the 2020 American Thoracic Society International Conference.

## Materials and methods

This single-center, retrospective cohort study at a single Veterans Affairs Medical Center included veterans admitted to an intensive care unit (ICU) who were diagnosed with septic shock from September 1, 2008, to August 31, 2018, and who received at least one dose of either cefepime or piperacillin-tazobactam for the initial antibiotic management of septic shock. The aim of this study was to determine whether the initial choice of antibiotic to manage septic shock was associated with differences in patient outcomes. Patient outcomes evaluated included AKI, CDI, hospital length of stay, and mortality. This study was approved by the University of Florida Institutional Review Board (approval number 201802352 ) and was conducted in accordance with protections for human subjects.

Septic shock was defined by clinical diagnosis of sepsis plus the receipt of an IV vasopressor (either norepinephrine, dopamine, epinephrine, or phenylephrine). The cohorts were divided based on whether they received cefepime or piperacillin-tazobactam for the initial antibiotic management. Veterans were excluded if they received both cefepime and piperacillin-tazobactam during the same admission, baseline serum creatinine levels were not collected, or secondary serum creatinine levels were not collected to assess a change from baseline. A sample size calculation was performed with the anticipated AKI incidence of 60% in the piperacillin-tazobactam group based on previous studies reporting this rate for septic shock patients [14]. An estimated 18% decrease in the incidence of AKI for the cefepime group was determined based on previous studies assuming a 15%-20% decrease in the incidence of AKI for critically ill patients on cefepime compared to piperacillin-tazobactam [6,8]. To detect a difference in the primary outcome with an alpha value of 0.05 and 80% power, a total of 240 patients (120 in each cohort) were needed for inclusion. Data were collected retrospectively utilizing the Data Access Request Tracker (DART) tool through the VA Informatics and Computing Infrastructure (VINCI).

The primary outcome of this study was AKI, defined by the Acute Kidney Injury Network (AKIN) guidelines. Per these guidelines, stage 1 AKI is defined as an increase in serum creatinine of >0.3 mg/dL or a 50%-99% increase in serum creatinine from baseline, stage 2 is defined as an increase in serum creatinine by 100%-199% from baseline, and stage 3 is defined as an increase in serum creatinine of >200% from baseline [15]. Baseline serum creatinine and highest serum creatinine during hospitalization were used to determine the development of AKI. Urine output measurements could not be assessed in this study due to the nature of the retrospective data collection. Secondary outcomes included CDI (defined by a positive *C. difficile* polymerase chain reaction test), hospital length of stay, ICU mortality and mortality within 30 days from hospital discharge.

For statistical analysis, the Mann-Whitney U test was used for continuous data (PlanetCalc V 3.0.3919.0; PlanetCalc, Moscow, Russia) and chi-square test was used for nominal data (GraphPad Software, San Diego, CA, USA). Additional statistical analysis was performed using Microsoft Excel 2018 (Microsoft, Redmond, WA, USA).

## Results

A total of 408 veterans received either piperacillin-tazobactam or cefepime, but not both, as the initial IV antibiotic management for septic shock at our institution in the defined study period and were screened for inclusion. Thirteen veterans were excluded for having incomplete baseline or secondary serum creatinine assessments. Of the remaining patients, 240 were randomly selected based on our anticipated sample size and for equally sized comparison groups (120 veterans in each cohort) for inclusion in the study. Baseline characteristics for these veterans are given in Table 1.

**Table 1:** Baseline characteristics of the included patients ICU: intensive care unit; IQR: interquartile range; IV: intravenous

	Piperacillin-tazobactam for septic shock (n=120)	Cefepime for septic shock (n=120)
Age (years), median [IQR]	68.0 [63.0, 75.3]	69.0 [63.0, 77.0]
Sex, male (%)	118 (98.3%)	116 (96.7%)
Baseline serum creatinine (mg/dL), median [IQR]	1.2 [0.9, 2.0]	1.5 [1.1, 2.5]
Concomitant IV vancomycin, n (%)	104 (86.7%)	107 (89.2%)
Medical ICU, n (%)	72 (60.0%)	93 (77.5%)
Surgical ICU, n (%)	31 (25.8%)	20 (16.7%)
Unspecified ICU, n (%)	17 (14.2%)	7 (5.8%)

The proportion of AKI in the cefepime group was 58.3% compared to 60.0% in the piperacillin-tazobactam group (p = 0.90). The proportion of stage 3 AKI was 17.1% in the cefepime group compared to 30.6% in the piperacillin-tazobactam group (p = 0.09), the proportion of stage 2 AKI was 28.6% in the cefepime group compared to 23.6% in the piperacillin-tazobactam group (p = 0.63), and the proportion of stage 1 AKI was 54.2% in the cefepime group compared to 45.8% in the piperacillin-tazobactam group (p = 0.40). The proportion of positive *C. difficile *polymerase chain reaction tests in the cefepime cohort was 5.8% compared to 5.0% in the piperacillin-tazobactam cohort (p = 0.74).

The median hospital length of stay in the cefepime cohort was 9.5 days (interquartile range [IQR] 4.0, 20.0 days) compared to 12.0 days (IQR 5.0, 22.3 days) in the piperacillin-tazobactam group (p = 0.15). The proportion of ICU mortality from any cause was 55.8% in the cefepime group compared to 37.5% in the piperacillin-tazobactam group (p < 0.01). The proportion of mortality within 30 days of hospital discharge was 65.8% in the cefepime group compared to 52.5% in the piperacillin-tazobactam group (p = 0.049). Results are summarized in Table 2.

**Table 2:** Results of the study AKI: acute kidney injury; ICU: intensive care unit; IQR: interquartile range

	Piperacillin-tazobactam (n=120)	Cefepime (n=120)	p-value
AKI, n (%)	72 (60.0%)	70 (58.3%)	0.90
Highest serum creatinine (mg/dL), median [IQR]	2.1 [1.4, 4.1]	2.6 [1.6, 4.3]	0.94
Clostridioides difficile, n (%)	6 (5.0%)	7 (5.8%)	0.74
Hospital length of stay (days), median [IQR]	12.0 [5.0, 22.3]	9.5 [4.0, 20.0]	0.15
ICU mortality, n (%)	45 (37.5%)	67 (55.8%)	<0.01
Mortality within 30 days of discharge, n (%)	63 (52.5%)	79 (65.8%)	0.049

## Discussion

This study comparing the use of piperacillin-tazobactam versus cefepime for the initial antibiotic management of septic shock found no significant differences in common adverse effects, but did find a significant difference in the observed mortality between cohorts. While these findings are noteworthy, further discussion of the data is warranted.

The increased mortality in the cefepime cohort was statistically significant. The previous literature directly comparing piperacillin-tazobactam to cefepime in the setting of septic shock does not report a difference in mortality between the two antimicrobial agents [9,10]. Of the patients admitted to the medical ICU (MICU), a higher percentage was initiated on cefepime than piperacillin-tazobactam. Thus, mortality may not have been influenced by the use of cefepime compared to an alternative agent, but representative of the severity of illness at MICU admission compared to surgical ICUs. Moreover, the source of infection may have differed between medical and surgical patients. For instance, surgical patients may have received piperacillin-tazobactam more frequently due to the nature of infections requiring surgical intervention and the need for anaerobic coverage whereas patients admitted to the MICU may have been treated more commonly for pneumonia or infections without an obvious source. The choice of agent may also have been influenced due to the changes in prescribing patterns after the publication of early literature reporting that the concomitant use of piperacillin-tazobactam and vancomycin increases the incidence of AKI [5,8].

Broad-spectrum antibiotics initiated in septic shock should target both gram-positive and gram-negative organisms. Vancomycin is commonly the drug of choice in septic shock for the coverage of resistant gram-positive organisms, such as methicillin-resistant *Staphylococcus aureus* (MRSA), although it has the potential to be nephrotoxic [16,17]. Nephrotoxicity is particularly concerning for patients in septic shock, as they have an increased risk of developing renal dysfunction. In this study, large majorities of both groups (89.2% in the cefepime group, 86.7% in the piperacillin-tazobactam group) similarly received concomitant vancomycin. Thus, it is unlikely that the use of vancomycin influenced the incidence of AKI in one group over the other. Sepsis guidelines recommend dual antipseudomonal coverage [1]. This commonly refers to the addition of an aminoglycoside or fluoroquinolone to an antipseudomonal beta-lactam. The use of aminoglycosides may further increase the risk of nephrotoxicity [18]. However, in this study, no patients received concomitant aminoglycosides. Our results did not show a significant association between the incidence of AKI and the choice of antibiotic for the initial management of septic shock, which is inconsistent with the previously published literature. Of note, the cefepime group had a higher median serum creatinine level at baseline that may have represented more the preexisting kidney disease and risk of AKI, but whether this impacted the results of this study is unknown. Based on previous studies evaluating concomitant piperacillin-tazobactam and vancomycin, we would have expected the piperacillin-tazobactam cohort to have a higher proportion of AKI compared to the cefepime cohort. However, our study was done in the critical care setting among an older population specifically with septic shock, which are risk factors for an increased incidence of AKI. Our study assessed patients regardless of their condition upon hospital admission or their type of infection; therefore, it is not known if these factors would have impacted the results. While we did see the 60% incidence of AKI in the piperacillin-tazobactam group as we anticipated, the incidence of AKI in the cefepime group was similar. It is possible a smaller difference than was anticipated could have been present, and therefore, the study was underpowered. Future studies would need to be higher powered in order to be able to detect the difference between these two cohorts.

Cefepime and piperacillin-tazobactam are commonly used for their broad-spectrum and antipseudomonal coverage, but these are only two of the several antibiotics used in the setting of sepsis. Empiric antibiotic therapy generally includes antipseudomonal agents in addition to an agent with activity against MRSA. As mentioned above, vancomycin is typically used as the agent to cover MRSA. The antipseudomonal agent may vary depending on the nature of the infection, allergies, resistance patterns, or other underlying conditions. Antipseudomonal beta-lactam antibiotics are typically preferred agents. While piperacillin-tazobactam and cefepime are the most commonly used antipseudomonal beta-lactams, others are available. Additionally, fluoroquinolones, including ciprofloxacin and levofloxacin, have activity against *Pseudomonas aeruginosa* and may be used as a second agent for dual-antipseudomonal coverage; however, the adverse effects such as QTc prolongation, hypoglycemia, and risk of tendon rupture limit the use of these medications for sepsis. Carbapenems are another class of antibiotics with antipseudomonal agents, but these medications are often reserved for resistant organisms as part of antimicrobial stewardship practices. This study did not evaluate for the use of other antipseudomonal agents, and thus, it is unknown how their use would have influenced the results. Current guidelines do not recommend a drug of choice when initiating empiric antimicrobial therapy because there have not been studies demonstrating superiority. Thus, this decision is based more on patient-specific factors and risk of adverse events.

The use of antibiotics is associated with CDI due to the disruption of normal gut microflora, therefore allowing the overgrowth of pathogenic organisms such as *C. difficile*. Both cefepime and piperacillin-tazobactam are broad-spectrum antibiotics, but cefepime has been associated with increased rates of CDI. A meta-analysis of antibiotics and the risk of CDI concluded that higher generations of cephalosporins carry an increased risk compared to piperacillin-tazobactam, which was found to be not statistically significant [11]. Piperacillin-tazobactam has been shown to have activity against *C. difficile* that prevents colonization, whereas cefepime and other cephalosporins do not have this activity [13]. Additionally, cephalosporins have different pharmacokinetic properties that allow them to reach higher concentrations within the gut, allowing for greater disruption of the normal gut flora. Within our septic shock population, the incidence of CDI was not statistically significant between cefepime and piperacillin-tazobactam.

The retrospective nature of this study poses a potential for the introduction of biases and the inability to control for confounders. Confounders present in this study that may have impacted the incidence of AKI included the use of concomitant nephrotoxic medications (e.g., intravenous contrast, loop diuretics, non-steroidal anti-inflammatory drugs), dose and duration of vasopressors, antibiotic dose and duration, use of corticosteroids during the course of therapy, proportion of mechanically ventilated patients, infection sites and organisms isolated, and a baseline severity of illness score to assess differences between the two groups. This study also had some important limitations due to the observational nature. Some degree of observer bias and selection bias is inevitable. The use of VINCI as our method of data collection limited the data able to be collected, including urine output and stool output to further assess AKI and CDI, mechanical ventilation status, baseline comorbid conditions (including chronic kidney disease or end-stage kidney disease), requirement for renal replacement therapy, and infection sites and organisms isolated. Additionally, the use of a single-center sample of predominantly older, male veterans limits the external validity and may not be applicable to other patient populations. While this study was appropriately powered with a sample size estimate, a larger sample size could have decreased type II error and allowed for the detection of smaller differences between groups.

## Conclusions

The choice of broad-spectrum antipseudomonal beta-lactam antibiotics for the initial management of septic shock appeared to have no impact on the incidence of the evaluated adverse effects, as no significant difference in AKI or CDI was observed. There was, however, higher mortality in the cefepime cohort compared to the piperacillin-tazobactam cohort that may warrant additional investigation. These results may assist clinicians in the treatment of patients with septic shock or help generate hypotheses for future studies. Further evaluation to determine the optimal approach to initial antibiotic management for patients with septic shock is necessary to enhance the quality of care and minimize adverse drug effects.
